# Brain morphometric changes in fibromyalgia and the impact of psychometric and clinical factors: a volumetric and diffusion-tensor imaging study

**DOI:** 10.1186/s13075-023-03064-0

**Published:** 2023-05-19

**Authors:** Benjamin Mosch, Verena Hagena, Stephan Herpertz, Martin Diers

**Affiliations:** Department of Psychosomatic Medicine and Psychotherapy, LWL University Hospital, Ruhr University Bochum, Alexandrinenstraße 1-3, 44791 Bochum, Germany

**Keywords:** Fibromyalgia, Pain, MRI, Brain morphometry, Voxel-based morphometry, Diffusion tensor imaging

## Abstract

**Background:**

Previous studies have repeatedly found distinct brain morphometric changes in patients with fibromyalgia (FM), mainly affecting gray and white matter abnormalities in areas related to sensory and affective pain processing. However, few studies have thus far linked different types of structural changes and not much is known about behavioral and clinical determinants that might influence the emergence and progression of such changes.

**Methods:**

We used voxel-based morphometry (VBM) and diffusion-tensor imaging (DTI) to detect regional patterns of (micro)structural gray (GM) and white matter (WM) alterations in 23 patients with FM compared to 21 healthy controls (HC), while considering the influence of demographic, psychometric, and clinical variables (age, symptom severity, pain duration, heat pain threshold, depression scores).

**Results:**

VBM and DTI revealed striking patterns of brain morphometric changes in FM patients. Bilateral middle temporal gyrus (MTG), parahippocampal gyrus, left dorsal anterior cingulate cortex (dACC), right putamen, right caudate nucleus, and left dorsolateral prefrontal cortex (DLPFC) showed significantly decreased GM volumes. In contrast, increased GM volume was observed in bilateral cerebellum and left thalamus. Beyond that, patients displayed microstructural changes of WM connectivity within the medial lemniscus, corpus callosum, and tracts surrounding and connecting the thalamus. Sensory-discriminative aspects of pain (pain severity, pain thresholds) primarily showed negative correlations with GM within bilateral putamen, pallidum, right midcingulate cortex (MCC), and multiple thalamic substructures, whereas the chronicity of pain was negatively correlated with GM volumes within right insular cortex and left rolandic operculum. Affective-motivational aspects of pain (depressive mood, general activity) were related to GM and FA values within bilateral putamen and thalamus.

**Conclusions:**

Our results suggest a variety of distinct structural brain changes in FM, particularly affecting areas involved in pain and emotion processing such as the thalamus, putamen, and insula.

**Supplementary Information:**

The online version contains supplementary material available at 10.1186/s13075-023-03064-0.

## Introduction

Fibromyalgia syndrome (hereinafter also referred to as “FM” for fibromyalgia) is a chronic pain disorder primarily characterized by widespread musculoskeletal pain that is oftentimes accompanied by a number of additional symptoms including cognitive, sleep and mood issues, fatigue, exhaustion, and an elevated risk for comorbidities [1, 2]. Patients with FM display an increased sensitivity (lower pain thresholds/higher pain ratings) to various pain modalities (thermal [3, 4], pressure [4, 5], electrical [6, 7], chemical [8, 9]). This phenomenon usually involves allodynia (perceived pain due to a non-painful stimulus) and hyperalgesia (increased pain from a painful stimulus) [10, 11].

The condition-specific combination of persistent physiological, cognitive, and affective symptoms (listed above), along with continuous psychological distress, leads to a significant reported reduction of the perceived quality of life [12, 13] and is highly disabling. However, at the current time, there is no universally recognized and effective treatment for FM and patients have a low probability of full recovery [14].

The heterogeneity and quantity of FM symptoms have led to a candid debate about the etiology and pathophysiological basis of the disorder, particularly concerning the neural background. Chronic pain disorders in general have repeatedly been shown to be associated with extensive structural, functional, and metabolic changes to the neural pain network of patients. In this context, particularly pronounced structural gray (GM) and white matter (WM) alterations have also been demonstrated in FM [15–20]. It is conceivable that the observed changes reflect cortical plasticity processes due to the chronically increased nociceptive input. Previous studies on GM volumetric outcomes have reported distinct alterations among FM patients, either in total volume [21, 22] or in specific brain areas, such as the thalamus or cerebellum [15, 17, 20]. On another note, several studies have found the extent of GM decreases in chronic pain disorders to be depending on the previous pain duration as well as to interact with the patients’ age [17, 22, 23].

With regard to WM changes in FM, Lutz et al. [19] demonstrated decreased fractional anisotropy (FA) in both thalami and insular regions, while reporting increased FA, inter alia, in the amygdalae, hippocampi, and anterior cingulate gyri. Decreased FA of the right thalamus was also observed in a different study by Sundgren et al. [24]. More recent investigations by Kim et al. [25] and Ceko et al. [17] found reduced FA values inside the corpus callosum, an area that is connected to bilateral sensorimotor cortex [25].

As seen from the results listed above, structural brain changes have been determined within a large number of different pain-related structures. However, a variety of methodological differences should be considered when comparing previous findings (e.g., data acquisition and analysis, heterogeneity of FM samples, different diagnostic criteria). Due to the diversity of symptoms experienced in FM, it poses a special challenge to isolate the specific effect that chronic pain has on patients’ brain morphometry.

In this study, we explored morphometric GM and WM brain changes in FM compared to healthy controls (HC) using magnetic resonance imaging (MRI) while considering the influence of various demographic, psychometric, behavioral, and clinical variables, involving age, symptom severity, pain duration, heat pain threshold, and depression scores. We performed voxel-based morphometry (VBM) on high-resolution T1-weighted MRI images in order to detect characteristic GM volumetric changes in FM. VBM is an analysis technique that comprises a voxel-wise measurement of focal differences in local concentrations of brain tissue. FM-specific WM changes were assessed using a diffusion tensor imaging (DTI) sequence that utilizes anisotropic diffusion to estimate the WM (axonal) organization of the brain.

We expected FM patients to display characteristic GM volumetric changes that have been reported in preceding investigation, primarily involving decreases in areas associated with pain and emotion processing, such as anterior cingulate cortex (ACC), amygdala, parahippocampal gyrus, insula, and prefrontal cortices, as well as increases within cerebellar structures [15, 19, 20, 22]. Additionally, we hypothesized that FM patients would exhibit distinct microstructural changes to their WM fibers, as continuous nociception and related stress have been shown to affect specific orientation-dependent aspects of WM connectivity [25, 26]. In view of our psychometric, behavioral, and clinical data, we expected pain severity, heat pain threshold, pain duration, depressive mood, and the general activity level to be associated with morphological brain changes in areas related to pain and emotion processing.

## Materials and methods

### Participants

Twenty-three female FM patients (aged 50.48 ± 9.89 years*,* range 32 to 68 years) and twenty-one female HC subjects (aged 46.62 ± 13.08 years*,* range 25 to 68 years) participated in the study. A two-sample *t*-test showed no statistically significant age difference between groups (*t*(42) = 1.1, *p* = 0.3). Left-handed persons, as assessed with the Edinburgh Handedness Inventory [27], were excluded. FM diagnoses were obtained by medical professionals and disorders fulfilled the criteria postulated by Wolfe et al. [2] (Fibromyalgia Survey Questionnaire (FSQ), see Table 1). FM patients were mainly recruited through social media support groups, whereas HC were recruited via newspaper announcements and face-to-face acquisition at several blood donation events of the German Red Cross. None of the tested participants had taken pain medication on the examination day. Furthermore, opioid use had been suspended no later than 3 days prior to the MRI session (2 cases: 1 × fentanyl patches, 1 × tramadol). We also excluded users of psychotropic medication, as well as psychotic patients and patients with an acute major depression and bipolar disorder. Sixteen FM patients reported previous major depressive episodes, four of whom also had a history of generalized anxiety disorder and/or PTSD as assessed with the structured clinical interview SKID-I [28]. None of the HC reported current or past psychopathological symptoms.

**Table 1:** Demographic, psychometric, and clinical data for FM and HC

	FM			HC		
	M	SD	Range	M	SD	Range
Age (years)	50.5	9.9	32–68	46.6	13.1	28–68
Pain duration in years	14.9	11.8	2–44			
CES-D	22.1	6.5	14–39	6.5	14–39	6–19
FIQ						
Physical functioning	1.4	0.6	0.2–2.4			
Total	60.2	17.6	19–88.2			
FSQ						
Symptom Severity Score	9.5	9.9	1–12			
Widespread Pain Index	10.8	3.7	6–19			
MPI						
Pain severity	4.0	15.8	6–19			
Interference	4.1	1.3	0.7–5.7			
Life control	3.4	1.3	0–6			
Affective distress	3.4	1.5	0–5.7			
Support	4.4	1.7	0–6			
Punishing responses	1.3	1.3	0–5.3			
Solicitous responses	3.8	1.8	0–6			
Distracting responses	3.4	1.4	0–5.7			
General activity level	7.2	2.4	2.6–11.5			

*FM* fibromyalgia, *HC* healthy controls, *M* mean, *SD* standard deviation, *CES-D* Center for Epidemiologic Studies Depression Scale, *FIQ* Fibromyalgia Impact Questionnaire, *FSQ* Fibromyalgia Survey Questionnaire, *MPI* West Haven-Yale Multidimensional Pain Inventory

The investigation took place at the Department of Neurology, Berufsgenossenschaftliches Universitätsklinikum Bergmannsheil, in Bochum between August 2019 and December 2020 and was approved by the ethics review board of the Medical Faculty Bochum, Ruhr University (15–5489). All participants gave written informed consent prior to participating in the study.

### Psychological questionnaire and physiological pain threshold assessment

FM patients completed the West Haven-Yale Multidimensional Pain Inventory (MPI) [29] (German version: Flor et al. [30]), the Fibromyalgia Impact Questionnaire (FIQ-G) [31], and the Fibromyalgia Survey Questionnaire (FSQ) [32]. In patients and controls, the presence of depression symptoms was assessed using the CES-D [33] (Center for Epidemiologic Studies Depression Scale; German version: ADS, Hautzinger and Bailer [34]). Additionally, the Edinburgh Handedness Inventory (EHI) [27] was assessed. Table 1 presents the demographic, psychometric, and clinical data for FM patients and HC. The structured clinical interview SKID-I [28] was conducted in all participants in order to rule out any acute mental illness.

Individual heat pain thresholds were determined using a 3 × 3 cm contact thermode (PATHWAY Pain & Sensory Evaluation System, Medoc Ltd. Advanced Medical System, Israel) and the software MEDOC Main Station 6.3. Ascending thermal stimulation cycles were presented on the participants’ left thenar and the mean value of the last three out of five consecutive thresholds was calculated. For heat pain threshold, subjects were asked to stop the stimulation via a mouse button when they started perceiving the stimulus as just painful.

### Clinical and behavioral data analysis

Clinical data and behavioral measures were analyzed using SPSS Version 26 for Windows (IBM Corp., 2019). Age in years, CES-D, and individual heat thresholds that were determined prior to the experiment (see the “Psychological questionnaire and physiological pain threshold assessment” section) were compared between HC and FM using two-sample *t*-tests.

### MRI data acquisition

MRI data were obtained on a Philips Achieva 3 T MRI scanner using a 32-channel standard head coil, packed with foam pads for fixation purposes. A high-resolution Magnetization Prepared Rapid Gradient (MPRAGE), comprising 204 sagittal slices, was obtained for each subject (TR = 6.98 ms, TE = 3.2 ms, flip angle 8°, 1 mm^3^ voxel size, FOV 256 × 204 mm^2^, acquisition time: 0.43 s). Diffusion-weighted images were acquired using a diffusion-weighted spin-echo (DwiSE) sequence (TR = 6.4 s, TE = 74.6 ms, *b*-value = 800 s/mm^2^, 2 mm^3^ voxel size, FOV = 224 × 120 mm^2^, acquisition time: 0.49 s) along 32 diffusion encoding directions. Additionally, one T2-weighted image with no diffusion (*b* = 0) was collected. All volumes were manually angulated in parallel to the AC–PC line and adjusted to include all frontal, central, parietal, and occipital cortical areas as well as upper parts of the temporal cortex and the cerebellum.

### Regions of interest

Relevant brain areas such as the thalamus, amygdala, putamen, pallidum, caudate nucleus, supplementary motor area (SMA), middle temporal gyrus (MTG), cerebellum, and insular, parahippocampal, prefrontal, orbitofrontal, cingulate, and primary and secondary somatosensory (SI, SII) cortices were defined based on the automated anatomical labeling (AAL) atlas 3 [35].

### Whole-brain volumetric analysis

High-resolution structural MPRAGE images were segmented in SPM using the Computational Anatomy Toolbox CAT12 (Structural Brain Mapping group [36], Jena University Hospital, Jena, Germany; http://dbm.neuro.uni-jena.de/cat/) (GM, WM, and cerebrospinal fluid (CSF)), normalized to Montreal Neurological Institute (MNI) standard space and smoothed with an isotropic Gaussian kernel of 8 mm FWHM. Total intracranial volume (TIV) was estimated as the sum of the three main brain tissue volumes (GM, WM, and CSF). Whole-brain group comparisons of cerebrospinal fluid (CSF), GM, and WM volumes as well as the total intracranial volume (TIV) were calculated in SPSS using two-sample independent *t*-tests (*p* < 0.05).

### Voxel-based morphometry: gray matter volume analysis

Group differences in local concentrations of brain tissue were assessed across the entire brain through voxel-based morphometry (VBM) which was implemented in SPM12 using CAT12. Segmented tissue class images were created in spatial correspondence to the template in MNI152NLin2009cAsym space. TIV was used as a covariate during the analyses in order to correct for different brain sizes. Age was also included as a covariate to take age-related gray matter changes into account. A two-sample *t*-test general linear model (GLM) was performed to assess brain volumetric differences between HC and FM. We applied a cluster-level correction of family-wise error (FWE) for multiple comparisons at a threshold of *p* < 0.05. In consideration of previous VBM studies showing volumetric changes of specific brain areas, we decided to perform further analyses using a less conservative uncorrected threshold of *p* < 0.001.

### Analysis of diffusion MRI data

Diffusion MRI was used to examine group differences of certain orientation-dependent aspects of brain tissue microstructure by analyzing the diffusion of water molecules. Diffusion MRI connectometry is a novel approach that allows tracking possible differences of WM tracts between groups as well as examining correlations of WM fibers with a variable of interest within the framework of a correlational tractography. Connectometry adopts a “tracking the correlation” paradigm that differs fundamentally from the conventional DTI analysis paradigm of finding the correlation between test variables and tract parameters. Within this framework, we used FA as the diffusion index of interest. FA is a scalar vector representing the directional selectivity of the random diffusion of water molecules, with higher FA pointing towards highly anisotropic water diffusion (e.g., heavily myelinated tracts) [37]. As reported by Yeh et al. [38], diffusion MRI connectometry can be more sensitive than conventional voxel-wise FA or APC mapping. However, it should be noted beforehand that FA changes cannot be interpreted in a linear fashion as changes of WM connectivity in a specific direction [39].

The connectometry was performed using DSI Studio (November 2020 build, Yeh et al. [40], http://dsi-studio.labsolver.org). The b-table was examined using an automated quality control routine to ensure its accuracy. Raw diffusion data were motion and eddy-current corrected using DSI Studio’s built-in preprocessing routine which is based on FMRIB Software Library’s (FSL) corresponding “eddy” tool. Corrected data were then reconstructed in the MNI space using Q-Space Diffeomorphic Reconstruction (QSDR) [41, 42]. The transformed distribution was used to obtain the spin distribution function (SDF) with a diffusion sampling length ratio of 1.25.

A correlational group tractography between FA and group (HC: 0; FM: 1) was carried out to compare regional FA values of HC and FM, using the participants’ age as a covariate. Correlational tractography has been shown to offer greater sensitivity than conventional tract- or voxel-based methods [40]. To map the different levels of correlation between the tracks and the group factor, different *t*-score thresholds of 2, 2.5, and 3 were used to visually study the tract-wise correlations at different cut-offs. In this regard, each threshold can be viewed as a different hypothesis. High *t* thresholds will map tracks with a stronger correlation effect, whereas lower *t* thresholds will map tracks with a weak correlation [40]. Models with a *t*-score threshold of 2 and a length threshold of 20 voxels yielded the strongest correlation while providing consistent results. The same parameters have been reported in a variety of previous connectometry studies using DSI Studio [38, 40, 43]. Topology-informed pruning with 4 iterations was implemented to filter the tracks and a total of 4000 randomized permutations were applied to obtain the null distribution of the track length. As suggested by the developer, a highly confirmative threshold of FDR = 0.05, corrected for the false discovery rate (FDR), was used to select tracts on a whole-brain level through a deterministic fiber tracking algorithm [44] to reveal all subcomponents of the fascicles that are significantly associated to our study variable group (FM vs HC). Yeh et al. [40] provide a detailed and complete description of diffusion MRI connectometry and the underlying methodology.

### Correlations of VBM and DTI data with clinical and behavioral measures

Regional relative GM volumes (corrected for different brain sizes) and FA values were correlated with demographic, psychometric, behavioral, and clinical variables using Pearson’s correlation coefficient (*r*) and a significance level of *p* < 0.05 in SPSS. In this analysis, we focused on brain regions that have displayed morphometric changes in our preceding analyses. The analyzed variables comprised three categories: (1) sensory-discriminative aspects of pain perception: pain severity (MPI) and heat pain threshold; (2)] chronicity: pain duration; (3) affective-motivational aspects of pain: depressive mood (CES-D) and the general activity level (MPI) (see Table 1).

## Results

### Clinical and behavioral characteristics of the participants

FM patients reported longstanding disease with a mean pain duration of 14.9 (± 11.8; range 2 to 44 years) years as well as high scores of pain severity (MPI: 4.0 ± 15.8), FM impact (FIQ: 60.2 ± 17.6), and depression (CES-D: 22.1 ± 6.5). The individual heat pain threshold determined prior to the experiment revealed no significant group differences between FM and HC, with a clear tendency towards lower values in FM (*mean* = 46.1) compared to HC (*mean* = 46.9) (*t*(41) = 1.6, *p* = 0.09).

### Whole-brain volumetric changes in FM patients

**Fig. 1:**
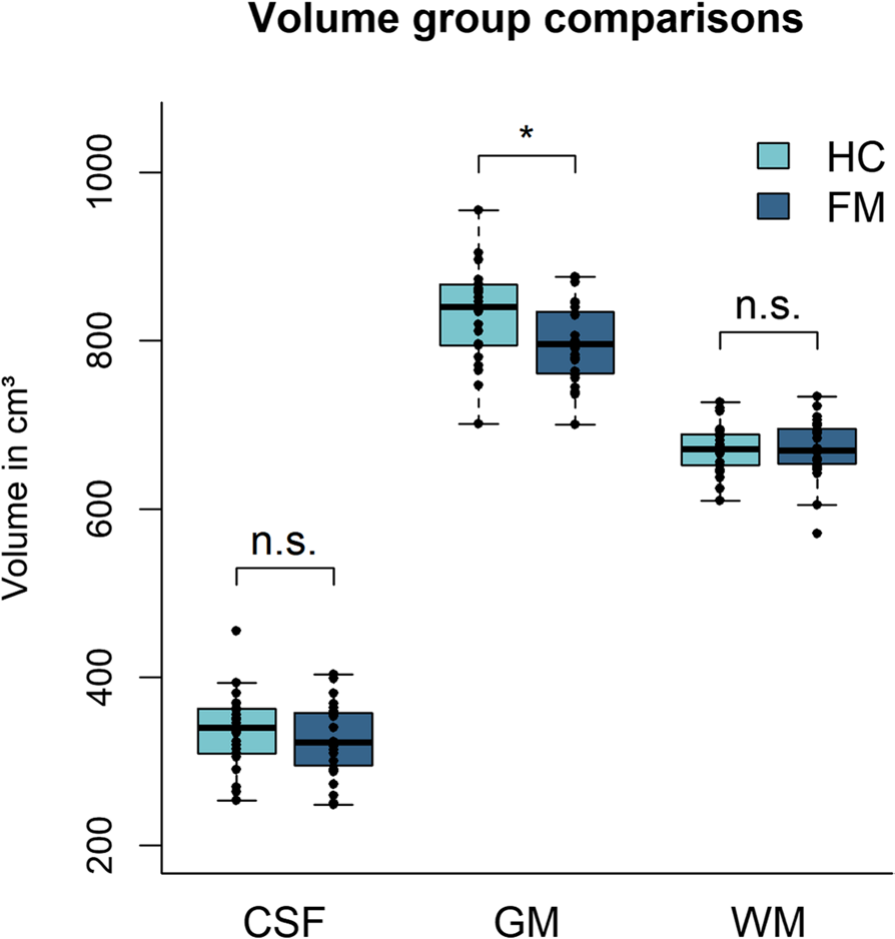
Whole-brain group comparisons of brain tissues (CSF, GM, and WM). Bar charts of the mean CSF, GM, and WM whole-brain tissue volumes (cm.^3^) for HC and FM. Individual GM mean values for each participant are indicated by black dots. CSF, cerebrospinal fluid; GM, gray matter; WM, white matter; HC, healthy controls; FM, patients with fibromyalgia; n.s., not significant; *p < .05

CSF as well as WM volumes did not differ significantly between FM and HC (CSF: *t*(43) = 0.9, *p* = 0.18; WM: *t*(42) = 0.13, *p* = 0.449). In contrast, GM volumes were shown to be significantly reduced in FM (*mean* = 797 ± 48 cm^3^) compared to HC (*mean* = 831 ± 61 cm^3^), *t*(40) = 2.1, *p* = 0.021 (see Fig. 1). Apart from this, FM patients (*mean* = 1793 ± 63 cm^3^) also displayed significantly decreased TIV compared to HC (*mean* = 1842 ± 63 cm^3^), *t*(43) = 2.7, *p* = 0.011.

### Regional GM volumetric changes in FM patients

**Fig. 2:**
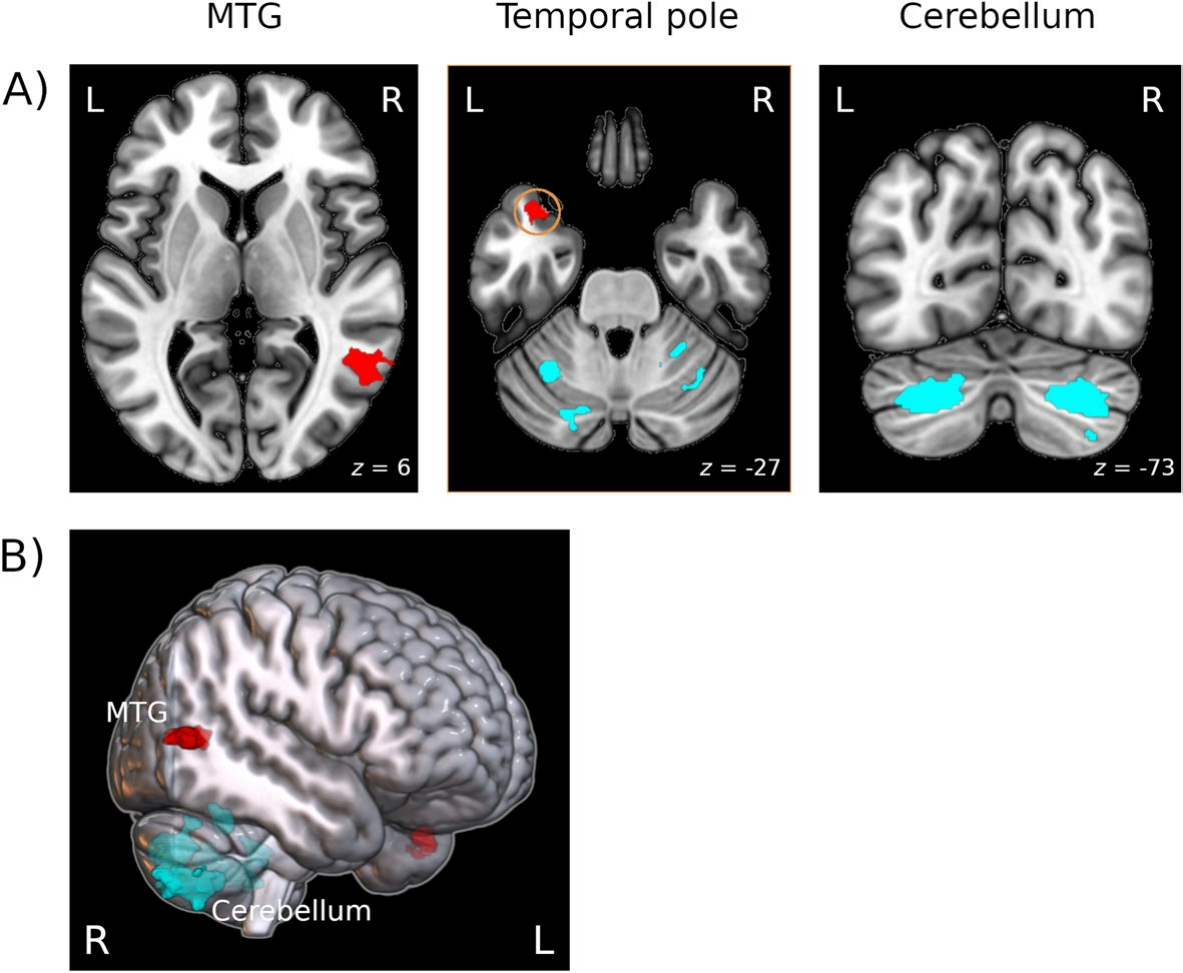
Brain regions with increased or decreased GM volumes in FM compared to HC. Structures that showed increased GM volume in FM are depicted in blue. Decreased GM volume is illustrated in red. **A** Axial and sagittal sectional images of the relevant clusters showing group differences. **B** 3D rendered illustration of the clusters. L, left; R, right; P, posterior; A, anterior; MTG, middle temporal gyrus. *p*(FWE) < .05

Group comparisons revealed FM-specific structural changes in a number of brain areas. When correcting for multiple comparisons (*p* < 0.05, FWE corrected), patients displayed decreased GM volumes in the left temporal pole (of the superior (STG) and middle temporal gyrus (MTG)) and right MTG. Significantly increased GM volume was found in major clusters within the bilateral cerebellum (Table 2 and Fig. 2).

**Table 2:** Brain regions showing significantly decreased or increased GM volume in FM compared to HC (*p* < 0.05, FWE corrected)

		MNI coordinates				
Comparison Brain region	Laterality	*x*	*y*	*z*	Cluster size (voxel)	*Z* score
HC > FM						
Temporal pole (STG/MTG)	L	− 36	14	− 27	193	4.8
MTG	R	54	− 60	6	365	4.7
FM > HC						
Cerebellum	R	27	− 80	− 38	2036	4.9
		26	− 48	− 26	191	3.8
	L	− 23	− 78	− 35	1489	4.7

*STG* superior temporal gyrus, *MTG* middle temporal gyrus, *L* left, *R* right, voxel size: 2.3 × 2.3 × 2.3 mm

Using a less conservative threshold (*p* < 0.001 uncorrected), we found a number of additional regional volumetric changes in FM. This involved decreased GM volumes in left MTG, right fusiform gyrus, parahippocampal gyrus, orbitofrontal cortex (OFC), right precentral cortex, right SMA, left dorsal anterior cingulate cortex (dACC), right putamen, right caudate nucleus, and left dorsolateral prefrontal cortex. On the other hand, further brain areas showing increased GM volumes in FM compared to HC involved left SMA, left thalamus, and right putamen (see Table S1).

### DTI connectometry results

One FM patient was excluded due to insufficient DTI data quality. Correlational group tractography for HC and FM revealed several tracts in which FA values were negatively correlated with the group parameter, indicating decreased FA and thus orientation-dependent changes to regional WM connectivity in FM (FDR < 0.05). These tracts involved left corticospinal tract, bilateral fornix, right corticospinal tract, bilateral superior corticostriatal tract, left cerebellum, right superior thalamic radiation, left arcuate fasciculus, left dentato-rubro-thalamic tract, and bilateral medial lemniscus and middle cerebellar peduncle.

**Fig. 3:**
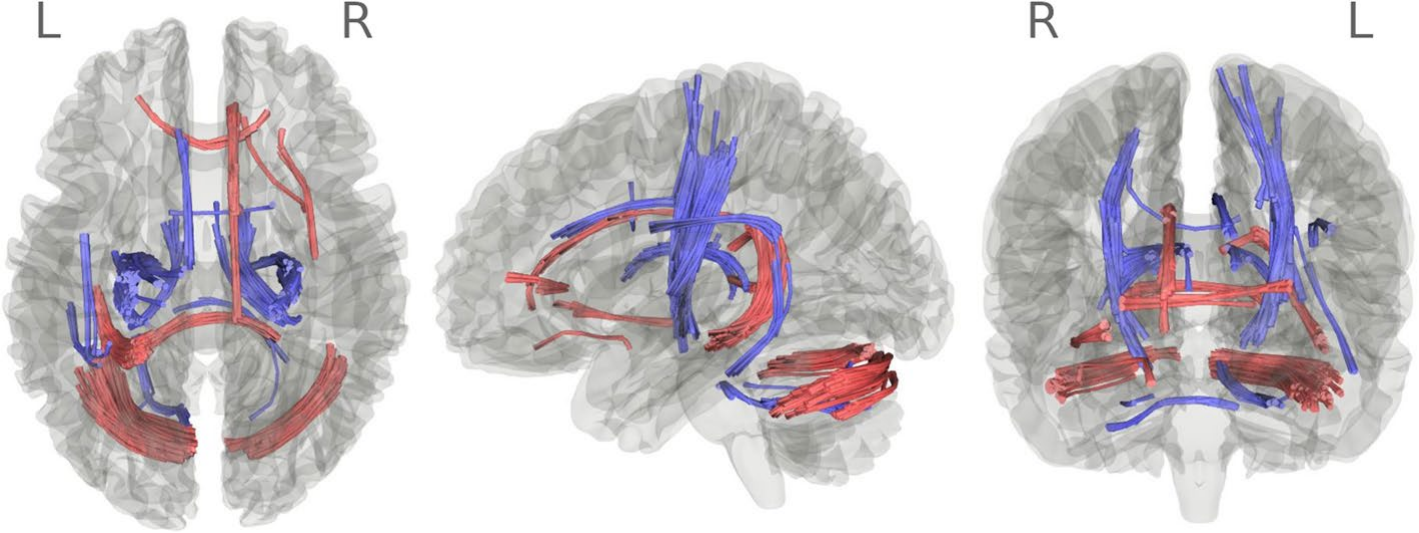
Correlational group tractography results. Comparison between fractional anisotropy (FA) positively (red) and negatively (blue) associated with the group parameter. *t* threshold: 2; length threshold: 20 voxels; permutation count: 4000, pruning iterations: 4; FDR < .05

On the other hand, a number of tracks displayed positive correlations with the group parameter, indicating increased FA in FM. Such correlations were found in right parolfactory cingulum, bilateral cerebellum, tapetum corporis callosi, forceps major and minor of the corpus callosum, and right inferior fronto-occipital fasciculus. WM tracts showing significant correlations with the group factor are illustrated in Fig. 3.

### Correlations with clinical and behavioral measures

**Fig. 4:**
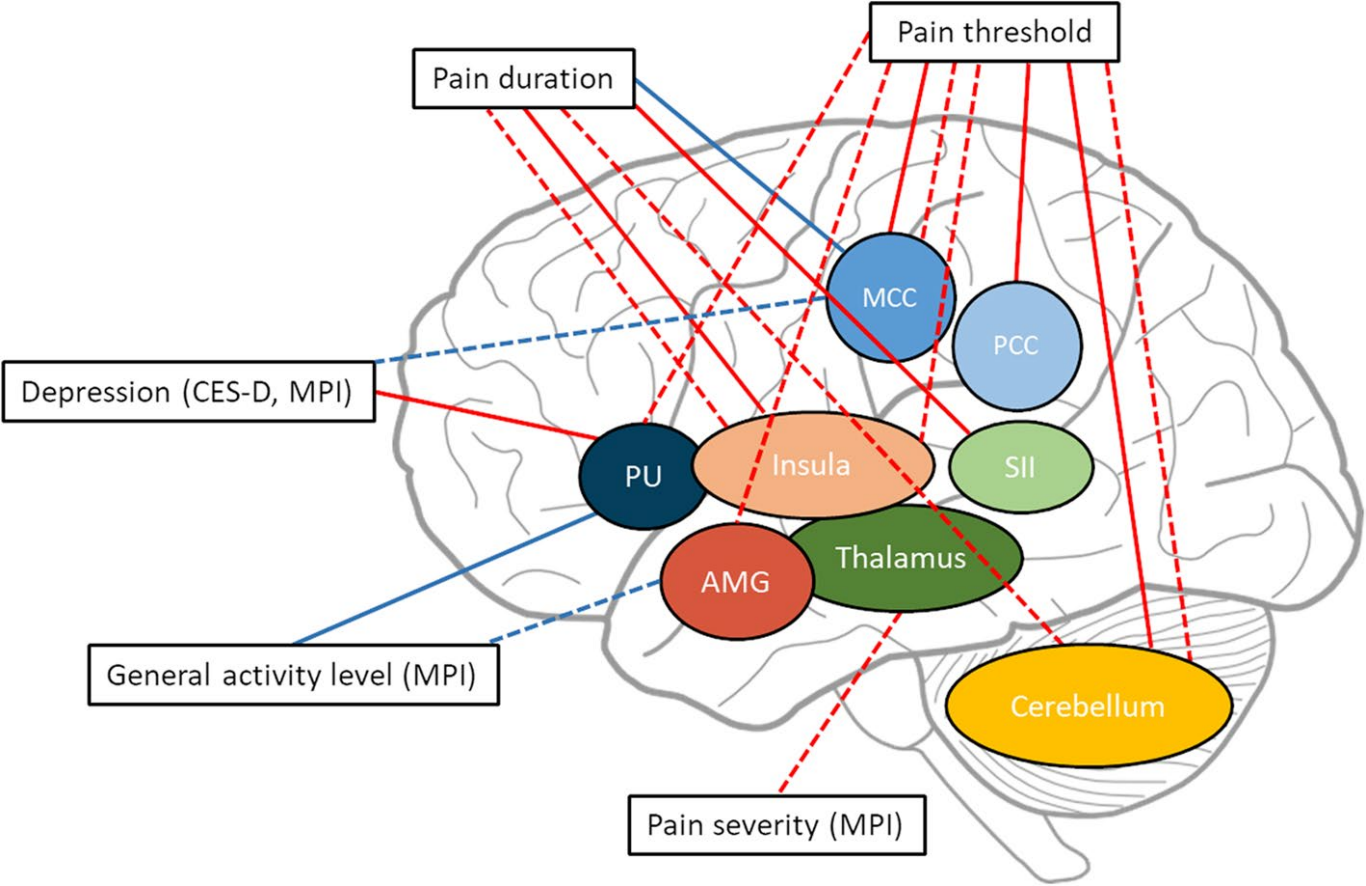
Brain regions that showed a significant correlation of GM or FA with behavioral/clinical data. Solid lines: correlations with GM volume; dashed lines: correlations with FA values; red lines: negative correlations; blue lines: positive correlations; AMG, amygdala; MCC, middle cingulate cortex; PCC, posterior cingulate cortex; SII, secondary somatosensory cortex; PU, putamen; PA, pallidum

Regarding sensory-discriminative aspects of pain perception, the MPI scale of pain severity was negatively correlated with FA values of multiple thalamic substructures, including ventral posterolateral as well as pulvinar anterior, pulvinar medial, pulvinar lateral, and pulvinar inferior thalamus (correlation coefficients *r* from 0.44 to 0.62, *p*-values from 0.048 to 0.003) (see Fig. 4). Additionally, the individual pain thresholds were negatively correlated with GM volumes of the right middle cingulate cortex (MCC) (*r* =  − 0.31, *p* = 0.039), bilateral posterior cingulate cortex (PCC) (L: *r* =  − 0.3, *p* = 0.046; R: *r* =  − 0.38, *p* = 0.01), and cerebellar lobule Crus II (*r* =  − 0.32, *p* = 0.037) as well as FA values of bilateral insula (L: *r* =  − 0.39, *p* = 0.01; R: *r* =  − 0.34; *p* = 0.029), right MCC (*r* =  − 0.36, *p* = 0.02), left amygdala (*r* =  − 0.37, *p* = 0.016), right putamen (*r* =  − 0.35, *p* = 0.024) and multiple cerebellar lobules (right Crus I, Crus II, Cerebellum 7b and 9; correlation coefficients *r* from − 0.31 to − 0.41*, p-*values from 0.045 to 0.01).

Concerning the chronicity, the duration of pain symptoms was negatively correlated with GM volumes of the right insular cortex (*r* =  − 0.43, *p* = 0.037) as well as left rolandic operculum (*r* =  − 0.5, *p* = 0.017) and positively correlated with right MCC (*r* = 0.41, *p* = 0.046). Moreover, chronicity showed negative correlations with FA values within the left insular cortex (*r* =  − 0.53, *p* = 0.036) and multiple cerebellar lobules (Crus I, Crus II, Cerebellum 3 and 6; correlation coefficients *r* from − 0.51 to − 0.64*, p-*values from 0.045 to 0.007).

Regarding affective-motivational aspects, depressive mood was negatively correlated with GM volumes of left putamen (CES-D: *r* =  − 0.31, *p* = 0.043), while the MPI scale of the general activity level was positively correlated with the structure (*r* = 0.39, *p* = 0.033). Furthermore, FA within the left MCC was positively correlated with CES-D scores (*r* = 0.31, *p* = 0.048) and bilateral FA values in the amygdala were positively correlated with the MPI scale of the general activity level (L: *r* = 0.45, p = 0.043; R: *r* = 0.5, *p* = 0.021).

## Discussion

The primary aim of the present investigation was to explore the precise form and extent of structural GM and WM changes related to FM as well as to explore possible correlations with clinical and behavioral measures. Group comparisons in fact demonstrated striking patterns of brain morphometric changes in FM patients compared to HC. In our initial analysis, we found whole-brain GM volumes as well as TIV to be significantly decreased in FM compared to HC. Similar findings have emerged in previous studies (e.g., Kuchinad et al. [22]) and support the notion of premature brain aging in patients with FM.

### Voxel-based morphometry

In addition to these general changes, we were particularly interested in the specific brain areas affected by the morphometric changes. For this purpose, we performed a VBM analysis that revealed distinct regional GM changes in our FM group. The most profound GM alterations were detected in the bilateral cerebellum. Increased cerebellar GM volume related to FM has been demonstrated in previous studies [45, 46]. Although the cerebellum plays a rather subordinate role in pain research, the area has repeatedly been shown to be an inherent part of the pain processing network and is strongly interconnected with the cerebral cortex [47, 48]. Beyond that, Kim et al. [49] compared FM and HC using covariance network analysis and found denser connections in the cerebellum as well as weaker connections in the frontal lobe of FM patients. The present study identified the most extensive abnormalities in Cerebellar lobules VIIb and Crus II, which are known to be associated with higher-order processes (e.g., executive, emotional, and cognitive) and lobule VIII, which is related to sensorimotor functions [50, 51].

Adopting a relatively conservative FWE correction for multiple comparisons, we demonstrated significantly decreased GM volumes of the left temporal pole (STG/MTG) and right MTG in FM. Decreased GM volume of the left MTG has recently been found by Sundermann et al. [52] in patients with FM and osteoarthritis. Accordingly, it may be argued that the group differences reported above could be related to chronic pain in general, rather than FM in particular. In this regard, decreased GM volume of the MTG has also been observed in patients with migraine [53], chronic myofascial pain [54], and trigeminal neuralgia [55]. Intriguingly, the area has recently been hypothesized to play a key role in redirecting attention away from pain and keeping involuntary thoughts about pain out of awareness [56]. In view of this, it is hardly surprising that decreased MTG volume (and altered functional connectivity of the area) was found to be associated with dysfunctional pain-related control processes and increased anticipatory anxiety [53, 57].

Most of the brain regions showing altered GM volumes when adopting a less conservative threshold of *p*_uncorr._ < 0.001 are significant components of the neural pain network, some of which have previously been demonstrated to be reduced in FM. More precisely, decreased GM volume or density has been reported in parahippocampal gyrus [22, 58], prefrontal cortex, and ACC [15, 17, 20]. As all of these structures are related to stress (parahippocampal gyrus) and pain processing, the observed changes might well be consequences of a long-term exposure to these symptoms. It should also be noted that prefrontal and cingulate cortices are generally ascribed pain modulatory and analgesic functions. Thus, GM atrophy in such areas could contribute to the maintenance of chronic pain symptoms in FM.

### Diffusion MRI connectometry analysis

FA is a parameter reflecting the directionality of water diffusion, or rather the degree to which the diffusion varies in different directions. However, due to a number of possible confounders contributing to metrics like FA, differences in scalar diffusion measures such as FA cannot be interpreted linearly as WM connectivity changes into a specific direction [39] (e.g., lower FA = low connectivity/tissue damage). Hereinafter, we therefore avoid making statements as to whether the observed changes amount, e.g., to possible decreases in WM integrity. Nevertheless, the regional microstructural WM group differences we detected provide valuable information about which areas and tracts show the most pronounced changes in FM.

To begin with, FM displayed decreased FA in bilateral medial lemniscus (also known as Reil’s band), a major ascending pathway consisting of heavily myelinated axons that is known to transmit tactile and proprioceptive information from the skin and joints to the thalamus and somatosensory cortex. In this way, the medial lemniscus plays a crucial role for processing of conscious proprioception, fine touch, and 2-point discrimination. Interestingly, FM patients have previously been found to show disruptions in all of these fields. For instance, FM has been linked to postural balance disorders and abnormalities of the proprioceptive system [59–61]. An example for altered sensations of fine touch in FM is the phenomenon of allodynia, a heightened sensitivity to non-nociceptive stimuli [62, 63]. Only recently, somatosensory temporal discrimination (STD), a measure for two-point discrimination, has been shown to be significantly prolonged in FM (in all extremities) [64]. The described findings clearly match the microstructural changes of WM connectivity (decreased FA) we found within the medial lemniscus of FM patients.

Interestingly, decreased FA was also found along tracts surrounding and connecting the thalamus, such as thalamic radiation and dentato-rubro-thalamic tracts, indicating changes to the area’s WM connectivity. This is of particular interest, as the thalamus is widely known to be critically involved in distributing and processing nociceptive information.

Further, a number of white matter tracts showing significantly increased FA in FM were located in and around the corpus callosum (tapetum corporis callosi, forceps major, and forceps minor of the corpus callosum), an area that is known to be strongly connected to bilateral sensorimotor cortex. Similar reductions of local FA values within the corpus callosum in FM have recently been described by Tu et al. [65] and Aster et al. [66]. On another note, the area is widely known to mediate interhemispheric transfer [67]. Thus, microstructural changes to the corpus callosum might consequently indicate a significant change to interhemispheric connectivity.

### Behavioral and clinical measures

Pain severity scores were negatively correlated with FA values in several thalamic substructures. These findings underline the significance of the observed changes in close relation with sensory-discriminative aspects of pain, such as the reported pain severity. On another note, we observed a significant negative correlation in FM between GM volume of the right insular cortex and the chronicity/duration of pain symptoms. This finding is in line with a number of previous investigations that reported a pronounced reduction of insular volume related to persisting chronic pain [68, 69]. GM volume of the left putamen was negatively associated with depressive symptoms, validating the previously postulated importance of the putamen in depressive disorders. In this context, Sacchet et al. [70] reported greater age-related volumetric decreases in the structure in patients with major depressive disorder. Consistently, we found GM volume of bilateral putamen to be positively correlated with the general activity level, representing an opposite pole to decreased activity levels that can typically be observed in depressive disorders. Accordingly, affective-motivational components of pain likewise play a decisive role in regard to the observed structural changes. This is further validated through the involvement of bilateral amygdala, which demonstrated FA positively correlated with the general activity level. Intriguingly, FA values of multiple pain-related ROIs were negatively correlated with the individual heat pain threshold, including bilateral insula, left amygdala, right MCC, right putamen, and multiple cerebellar lobules. Accordingly, increased pain sensitivity was found to be associated with greater FA within the examined ROIs of the pain network.

### Limitations

As noted earlier, some methodological characteristics of this study need to be considered when interpreting the results. In this regard, the informative value of diffusion indices such as FA is a critical factor. Regional anisotropy can be influenced by a number of confounding variables (e.g., axon diameter, packing density, and number of axons) [71]. Due to these and other reasons, differences in FA indicate some form of change to microstructural WM connectivity, but cannot be linearly interpreted as WM integrity changes into a certain direction [39]. However, we have attempted to circumvent this issue through our cautious interpretation of the findings.

Another limitation that should be mentioned is associated to the ROI-wise approach we have picked to investigate the relationship of regional brain morphometry and behavioral measures. Arguably, this procedure can in some cases lead to partial volume effects. However, we have decided to implement a ROI-wise approach, in addition to our WM tractography, as similar approaches have been used in numerous investigations on chronic pain on which we have based our study design thematically and methodically [17, 19, 24, 72]. Furthermore, we found a ROI-wise approach to be the most suitable way for us to investigate the correlations described above, as our goal was to explore possible associations of different regional microstructural characteristics with behavioral and clinical data. The consistent ROI-wise approach entails comparability of regional GM volumetric and diffusion data.

In general, our investigation was designed as an initial exploration of the topic described above, providing clues to plan possible follow-up studies. This context explains our exploratory approach and should also be taken into account when interpreting the results.

## Conclusions

The described findings delineate major structural brain changes in FM, affecting large parts of the neural pain network. Arguably, some of the most distinct FM-related regional morphometric changes were found in the thalamus, putamen, and insula, involving significantly reduced FA values that were related to the subjectively perceived pain severity as well as GM decreases. However, such morphometric changes have repeatedly been shown to be reversible after cessation of pain [73, 74] and might represent a temporary consequence of chronic nociceptive input, rather than permanent brain damage. This is also supported by the fact that we found positive correlations of GM in bilateral putamen with the general activity level, which could serve as an example for possible control strategies to reverse maladaptive neural changes.

## Supplementary Information

**Additional file 1:** **Table S1. **Brain regions showing significantly greater GM volume in HC compared to FM and vice versa (*p* ≤ 0.001 uncorrected).

## Data Availability

Further information and requests for resources and reagents should be directed to and will be fulfilled by the lead contact, Martin Diers (martin.diers@rub.de).

## Abbreviations

AAL: Automatic anatomical labeling atlas
CSF: Cerebrospinal fluid
DTI: Diffusion-tensor imaging
FA: Fractional anisotropy
FDR: False discovery rate
FM: Fibromyalgia
FWE: Family-wise error
GM: Gray matter
HC: Healthy controls
MRI: Magnetic resonance imaging
ROI: Region of interest
TIV: Total intracranial volume
VBM: Voxel-based morphometry
WM: White matter
