# Correction: Brain morphometric changes in fibromyalgia and the impact of psychometric and clinical factors: a volumetric and diffusion‑tensor imaging study

**DOI:** 10.1186/s13075-023-03077-9

**Published:** 2023-05-31

**Authors:** Benjamin Mosch, Verena Hagena, Stephan Herpertz, Martin Diers

**Affiliations:** Department of Psychosomatic Medicine and Psychotherapy, LWL University Hospital, Ruhr University Bochum, Alexandrinenstraße 1-3, 44791 Bochum, Germany

**Correction: Arthritis Res Ther 25, 81 (2023)**

**https://doi.org/10.1186/s13075-023-03064-0**

Following publication of the original article [1], the authors reported the department mentioned in affiliation 1 (Clinical and Experimental Behavioral Medicine) is a subordinate part of affiliation 2 (Department of Psychosomatic Medicine and Psychotherapy, LWL University Hospital, Ruhr University Bochum, Alexandrinenstraße 1‑3, 44,791 Bochum, Germany) and requested affiliation 1 to be removed.

The original article [1] has been updated.
