# Supplementary material for: Brain morphometric changes in fibromyalgia and the impact of psychometric and clinical factors: a volumetric and diffusion-tensor imaging study
Source: Arthritis Res Ther. 2023 May 19;25:81. doi: 10.1186/s13075-023-03064-0 (PMC10197341; doi:10.1186/s13075-023-03064-0)
Supplement: Supplementary file 1 — Additional file 1: Table S1. Brain regions showing significantly greater GM volume in HC compared to FM and vice versa (p ≤ 0.001 uncorrected). [file 13075_2023_3064_MOESM1_ESM.docx]

**Supplementary material**

**Table S1.**

Brain regions showing significantly greater GM volume in HC compared to FM and vice versa (p ≤ 0.001 uncorrected)

|  |  |  | MNI coordinates | | |  |  |
| --- | --- | --- | --- | --- | --- | --- | --- |
| **Contrast**  Brain region | Laterality | BA | *x* | *y* | *z* | Cluster size^a^ | *Z* score |
| **HC > FM** |  |  |  |  |  |  |  |
| Temporal pole | L | 38 | -36 | 14 | -27 | 193 | 4.79 |
|  | L | 28 | -23 | 6 | -27 | 12 | 3.58 |
|  | L |  | -45 | 8 | -38 | 3 | 3.3 |
|  | L |  | -57 | 5 | 0 | 4 | 3.22 |
|  | R | 38 | 27 | 9 | -27 | 56 | 3.81 |
|  | R | 38 | 39 | 5 | -30 | 23 | 3.63 |
| Middle temporal gyrus | R | 37 | 54 | -60 | 6 | 365 | 4.74 |
|  | R | 21 | 71 | -42 | -8 | 55 | 4.3 |
|  | R |  | 60 | -3 | -21 | 47 | 3.56 |
|  | R | 37 | 57 | -68 | 0 | 5 | 3.26 |
|  | R |  | 59 | -65 | 15 | 7 | 3.22 |
|  | L |  | -60 | -57 | 5 | 66 | 4.23 |
|  | L |  | -50 | -53 | -2 | 11 | 3.33 |
| Fusiform gyrus | R |  | 47 | -36 | -26 | 35 | 3.95 |
| Parahippocampal gyrus | L | 34 | -12 | -2 | -26 | 20 | 3.89 |
|  | R | 34 | 15 | -8 | -24 | 3 | 3.2 |
| OFC | L |  | -23 | 14 | -15 | 7 | 3.68 |
| Precentral cortex | R | 4 | 33 | -21 | 54 | 36 | 3.63 |
|  | R | 4 | 18 | -32 | 66 | 3 | 3.34 |
| Inferior temporal gyrus | L | 21 | -60 | -41 | -18 | 21 | 3.7 |
|  | R |  | 51 | -44 | -11 | 12 | 3.53 |
|  | R |  | 47 | -11 | -29 | 6 | 3.35 |
| SMA | R | 32 | 9 | 11 | 56 | 4 | 3.5 |
|  | R |  | 5 | 17 | 47 | 20 | 3.38 |
| Superior frontal cortex | L |  | -15 | 27 | 63 | 5 | 3.3 |
|  | L |  | -18 | 21 | 39 | 38 | 3.74 |
| Inferior frontal gyrus | L |  | -38 | 17 | -17 | 7 | 3.62 |
| Superior frontal gyrus | R |  | 14 | 41 | -20 | 13 | 3.55 |
| Middle frontal gyrus | R |  | 39 | 53 | -9 | 7 | 3.54 |
| Temporal pole / rolandic  operculum | L | 22 | -57 | 5 | 0 | 51 | 3.27 |
| dACC | L | 32 | -11 | 15 | 32 | 12 | 3.32 |
|  | L | 32 | -11 | 42 | 15 | 3 | 3.29 |
| Putamen | R |  | 24 | 21 | -9 | 5 | 3.15 |
| Supramarginal gyrus | L | 40 | -59 | -41 | 47 | 22 | 3.15 |
| Caudate nucleus | R |  | 9 | 18 | -9 | 12 | 3.14 |
| DLPFC | L | 9 | -27 | 32 | 42 | 4 | 3.25 |
|  |  | 8 | -27 | 27 | 44 | 1 | 3.33 |
| MCC | R |  | 11 | -36 | 42 | 3 | 3.29 |
|  |  |  |  |  |  |  |  |
| **FM > HC** |  |  |  |  |  |  |  |
| Cerebellum Crus II | R |  | 27 | -80 | -38 | 2036 | 4.88 |
| Cerebellum VIIb | R |  | 42 | -62 | -54 |  | 4.78 |
| Cerebellum VIII | R |  | 42 | -53 | -54 |  | 4.68 |
| SMA | L |  | -11 | 23 | 68 | 133 | 4.71 |
| Cerebellum | L |  |  |  |  |  |  |
| Cerebellum Crus II | L |  | -23 | -78 | -35 | 1489 | 4.69 |
| Thalamus | L |  | -11 | -11 | 2 | 14 | 3.88 |
| Fusiform | L |  | -24 | -66 | -14 | 29 | 3.84 |
| Cerebellum IV V | R |  | 23 | -42 | -24 | 107 | 3.82 |
| Cerebellum III | L |  | -14 | -32 | -24 | 57 | 3.81 |
|  | L |  | -8 | -38 | -15 | 14 | 3.7 |
| Cerebellum VI | R |  | 26 | -48 | -26 | 191 | 3.77 |
|  | L |  | -17 | -66 | -27 | 18 | 3.58 |
| Putamen | R |  | 24 | -3 | 14 | 9 | 3.42 |
| Postcentral | R |  | 53 | -6 | 30 | 6 | 3.41 |

Note. BA: Brodmann area; L: left; R: right; OFC: orbitofrontal cortex; SMA: supplementary motor area; dACC: dorsal anterior cingulate cortex; DLPFC: dorsolateral prefrontal cortex; MCC: midcingulate cortex; voxel size: 2.3 x 2.3 x 2.3 mm; ^a^ in voxel.
